# Mapping hydrologic alteration and ecological consequences in stream reaches of the conterminous United States

**DOI:** 10.1038/s41597-022-01566-1

**Published:** 2022-07-28

**Authors:** Ryan A. McManamay, Rob George, Ryan R. Morrison, Benjamin L. Ruddell

**Affiliations:** 1 Department of Environmental Science, Baylor University, Waco, TX 76798 USA; 2 School of Informatics, Computing and Cyber Systems, Northern Arizona University, Flagstaff, AZ 86011 USA; 3 Department of Civil and Environmental Engineering, Colorado State University, Fort Collins, CO 80523 USA

**Keywords:** Hydrology, Freshwater ecology, Biogeography

## Abstract

Environmental flows are critical for balancing societal water needs with that of riverine ecosystems; however, data limitations often hinder the development of predictive relationships between anthropogenic modifications to streamflow regimes and ecological responses – these relationships are the basis for setting regional water policy standards for rivers. Herein, we present and describe a comprehensive dataset of modeled hydrologic alteration and consequences for native fish biodiversity, both mapped at the stream-reach resolution for the conterminous U.S. Using empirical observations of reference conditions and anthropogenically altered streamflow at over 7000 stream gauges, we developed a predictive model of hydrologic alteration, which was extended to >2.6 million stream reaches. We then used a previous nationwide assessment of ecological responses to hydrologic alteration to predict fish biodiversity loss in stream reaches resulting from streamflow modification. Validation efforts suggested hydrologic alteration models had satisfactory performance, whereas modeled ecological responses were susceptible to compounded errors. The dataset could ameliorate regional data deficits for setting environmental flow standards while providing tools for prioritizing streamflow protection or restoration.

## Abstract (summary)

**Table:** 

Measurement(s)	hydrologic alteration, human alteration of streamflow regimes • fish biodiversity
Technology Type(s)	Random forest • Quantile regression techniques
Factor Type(s)	human disturbances in streams (land use, dam storage, water use)
Sample Characteristic - Organism	Freshwater fish
Sample Characteristic - Environment	stream
Sample Characteristic - Location	contiguous United States of America

## Background & Summary

Freshwater ecosystems are facing a global biodiversity crisis^1^. Despite only 0.01% of the Earth’s surface water occurring as freshwater ecosystems, these systems harbor 7% of the world’s described species and one third of all vertebrates^2,3^. Yet these same ecosystems are facing alarming rates of habitat and biodiversity loss. Even among freshwater ecosystems, rivers and streams are considered numerically rare despite providing disproportionate services to society, ultimately leading to their over-exploitation^4^. Much of the exploitation of lotic systems is directly related to the manipulation of natural streaflow variability. For instance, roughly 50% of rivers and streams across the world are hydrologically altered from their natural state^5,6^, with as much as 80% of streams in Canada^7^ and 80% of streams in the conterminous US^8^ displaying signs of alteration in streamflow regimes. By 2050, climate change is expected to have altered streamflow regimes in as much as 80% of the global terrestrial land surface aside from that already altered from human infrastructures^9,10^ - only exacerbating decades of declines in freshwater fauna populations^11^. The monumental challenge faced by modern society is “bending the curve” of freshwater biodiversity loss, all while addressing climate change mitigation and human population growth^1^.

In their Emergency Recovery Plan for dampening or reversing declines in freshwater biodiversity, Tickner *et al*.^1^ lay out several major actions, the first being the widespread implementation of environmental flows, i.e., the quantity and timing of streamflow volumes required to sustain the ecological integrity of river systems. With competing demands for limited water quantities, the inevitable question that follows is “How much water do river [ecosystems] need?”^12,13^. Prescribing environmental flows for rivers requires understanding dependencies or linkages between essential stream flow components (e.g., magnitude, duration, frequency of flows) and ecological processes. Even more critical is understanding ecological responses to human-induced alterations to naturally variant streamflow regimes – generally termed “flow-ecology relationships”^14^. At a regional level, flow-ecology relationships, especially thresholds or tipping points of streamflow alteration, can be used as general guidelines for environmental flow implementation and preventatives for over-extraction – this general process has been formalized as the Ecological Limits of Hydrologic Alteration (ELOHA) framework^15^. The ELOHA framework has been implemented in at least 20 US states and several countries^16^; however, a practical demand of developing empirical flow-ecology relationships include identifying datasets with sufficient empirical observations. A commonly reported shortcoming of regional ELOHA studies is the limited availability of information to support the development of flow-ecology relationships, either from the scientific literature^14^ or from pre-existing inventories of biological surveys and hydrologic records^17^.

Imposing restrictive criteria on observation datasets dramatically reduces sample sizes and generalizations that can be drawn from the data. For example, in a nationwide study evaluating flow-ecology relationships, only 237 (3%) of 7,000 US Geological Survey gaging locations had co-occurring macroinvertebrate or fish sampling surveys^8^. One solution to ameliorate the data-shortage issue is to increase the volume and density of systematic biological surveys; however, US federal and state-funded systematic biological surveys are already considered regionally comprehensive (>1.5 million sampling events) yet provide high-quality survey data for only 13% of US streams^18^. Even marginal increases in the percentage of surveyed streams would come at great cost to resources. A more economical solution is to model expected hydrologic and ecological conditions in ungauged stream reaches to fully leverage the wealth of existing hydrologic and biological data^19^. For instance, George *et al*.^17^ used modeled estimates of hydrologic conditions in streams to yield a nationwide flow-ecology dataset of 6,452 observations – a value almost 9 times the observations reported in a comparable study relying on overlapping biological surveys and hydrological data^20^. Obviously, a limitation of such an approach is the uncertainty of modeled versus observed hydrologic conditions and subsequent error propagation that arises in developing flow-ecology relationships^19^.

Given the importance of ensuring region-wide extensions of the ELOHA framework, especially in data-limited regions, a comprehensive dataset of modeled hydrologic conditions or hydrologic alteration values and ecological consequences, mapped to the stream-reach resolution, would be valuable to the research and practitioner community. Not only could such a dataset address limitations of contemporary flow-ecology relationship development, but it expedites the entire 4-step scientific components embodied within the ELOHA process and provides substantial time-savings for researchers and managers. Herein, we present a dataset of modeled hydrologic (streamflow) alteration, summarized in 43 metrics, and resulting estimates of losses in fish species richness for 2.6 million stream reaches within the conterminous US.

## Methods

### Overview of hydrologic and ecological mapping protocol

**Fig. 1:**
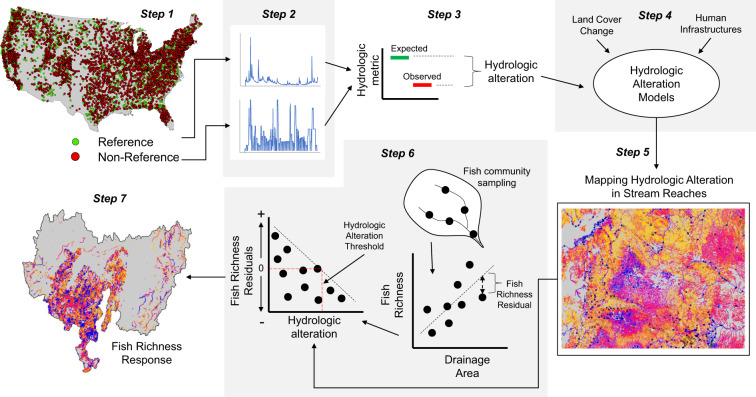
Overview of the 7-step approach used to map hydrologic alteration and ecological consequences in stream reaches of the conterminous US.

Mapping hydrologic and ecological alteration at the stream reach level followed a 7-step process that builds upon several previously published methods (Fig. 1). The steps include: (1) compiling a nationwide dataset of streamflow gauges from the US Geological Survey (USGS) and distinguishing reference and non-reference gages and associated records^21–23^, (2) assembling stream flow records and calculating hydrologic indices^23^, (3) quantifying hydrologic alteration for stream gages^22^, (4) developing models to predict hydrologic alteration from human disturbance variables^24^, (5) using models to extrapolate hydrologic alteration to ungauged stream reaches^24^, (6) developing empirical models of fish species richness responses to hydrologic alteration^17^, and (7) mapping fish richness responses to ungauged stream reaches based on modeled estimates of hydrologic alteration. Methodological details are provided in each of the publications cited above; however, an overview of the steps is provided here. We elaborate more fully on the detailed methodology starting at step 3, as this reflects more of the focus of the technical validation of the dataset (Fig. 1).

#### Step 1 - Compiling a nationwide streamflow dataset

We assembled streamflow information for 7,088 USGS stream gages with at least 15 years of daily discharge data as of 2010. We only included gages with at least 15 years of complete annual records (i.e., those with <= 30 days of missing daily data). The influence of climate variability on hydrologic statistic values dampens as periods of records increase, and typically, at least 15 years of record are required to stabilize variation in indices, at least to acceptable levels for spatial analyses^25^. However, a noted limitation of our analysis is that we did not formally control for climate variation in the calculation of hydrologic statistics from stream records varying in length and duration, as compared to Eng *et al*.^26^. However, Eng *et al*.^26^ found that climate variability had minimal influence on hydrologic alteration at stream gages relative to human land and water management.

Gages were partitioned into reference (n = 2,249) and non-reference (n = 4,839) condition based on geographical evaluations of human disturbance regimes in basins upstream of each gage, reviews of USGS water reports, and visually inspecting flow duration curves^21,22^. For ease of terminology, we use the term “reference” to indicate the “least disturbance” condition for a region, as defined by Stoddard *et al*.^27^. For streams currently regulated by dams and with at least 15-yr records extending prior to dam regulation (n = 250), streamflow records were partitioned into pre- and post-dam construction time periods^22^. Except for pre-dam construction records, periods of record for reference conditions displayed considerable temporal overlap (at least 50% of overlap in records)^23^, as did periods of record for non-reference conditions^22^.

#### Step 2- Assembling streamflow records and calculating hydrologic statistics

Daily streamflow records were obtained from the USGS National Water Information System (NWIS) website (https://waterdata.usgs.gov/nwis). For reference streams, the entire period of record was used for calculating hydrologic statistics, whereas for non-reference gages, only periods overlapping with contemporary human disturbance regimes were used (1980–2010) due to the temporal limitations of anthropogenic disturbance variables used to predict hydrologic alteration. The National Hydrologic Assessment Tool (www.sciencebase.gov/catalog/item/5387735ee4b0aa26cd7b5461) was used to calculate 110 hydrologic statistics summarizing the magnitude, frequency, duration, timing, and rate of change in flow for all reference and non-reference stream flow records^23^.

#### Step 3- Calculating hydrologic alteration indices at gages

Calculating hydrologic alteration at non-reference gages first required estimating reference or natural hydrologic conditions as a baseline from which the degree of alteration could be assessed. Of the 110 hydrologic statistics above, we selected 41 indices that adequately represent the multi-dimensional nature of regional variation in hydrologic across the US^23^ and have been used in previous assessments of hydrologic alteration^22^ (Table 1). These 41 indices include the Indicators of Hydrologic Alteration^12^, a series of non-redundant metrics representing the predominant variation embodied by almost 200 hydrologic variables^28^. Since the 41 indices are univariate summaries of hydrologic conditions, two additional indices, a hydrologic alteration index and a seasonality alteration index, were calculated to represent multivariate impacts to overall variation among hydrologic metrics and the distribution of monthly flows, respectively (more details provided in Step 3 expanded).

**Table 1:** Hydrologic indices used in the study and their description. Table taken directly from George *et al*.^17^.

Index	Definition
Magnitude of flow events	
MA1	Mean Daily Flow
MA2	Median Daily flow
MA3	Variability in daily flows
MA12–23	Mean monthly flow for all months, January (12) through December (23)
MA41	Mean annual runoff
ML17	Baseflow 1. Seven-day minimum flow divided by mean annual daily flows averaged across all years.
ML19	Baseflow 2. Mean of ratio of the lowest annual daily flow to the mean annual daily flow times 100 averaged across all years
MH20	Mean annual maximum flows divided by catchment area
Duration of flow events	
DL1–5	Magnitude of minimum annual flow for 1-/3-/7-/30-/90-day means
DL16	Low flow pulse duration
DL18	Number of zero-flow days
DH1–5	Annual maxima of 1-/3-/7-/30-/90-day means of daily discharge
DH15	High flow pulse duration
Frequency of flow events	
FL1	Low flow pulse count. Number of annual occurrences during which the magnitude of flow remains below a lower threshold.
FH1	High flood pulse count. See FL1.
FH6	Flood frequency. Mean number of high flow events per year using 3 times median annual flow
FH7	Flood frequency. Mean number of high flow events per year using 7 times median annual flow
Timing of Flow Events	
TA1	Constancy
TA2	Predictability of flow
Rate of Change of flow events	
RA1	Rise rate. Mean rate of positive changes in flow from one day to the next.
RA3	Fall Rate. Mean rate of negative changes in flow from one day to next.
RA8	Reversals. Number of positive and negative changes in water conditions from one day to the next.
Cumulative measures	
HAI	Hydrologic Alteration Index. A measure of cumulative hydrologic change of the most important dimensions of the flow regime. See Methods.
Seasonality	Seasonality alteration. A cumulative measure of departures in mean monthly flows for all months. See Methods.

With the exception of cumulative indices, index codes are taken from Olden *et al*.^28^.

Generally, reference condition values of hydrologic indices were estimated for non-reference gages using random forest statistical models constructed from reference gauges or gauges with pre-dam hydrologic records^22^. Random forest model performance was high with a median variance explained of 91% among all hydrologic indices and median normalized RMSE of 0.513^22^ (normalized RMSE by range of values). In cases where statistical models were unreliable (i.e., indices depicting timing of low and high flows), non-reference stream gages were assigned to a hydrologic class representing a range of reference condition index values^22^. In these situations, the 90^th^ percent confidence interval of hydrologic index values represented by all reference gauges within a hydrologic class was used to represent the reference flow condition. Observed hydrologic indices were then compared to estimated reference conditions to calculate hydrologic alteration indices, characterizing the degree of changes in stream flow due to human influence (see next section).

#### Step 4 and 5 - Predicting hydrologic alteration and mapping to U.S. streams

**Fig. 2:**
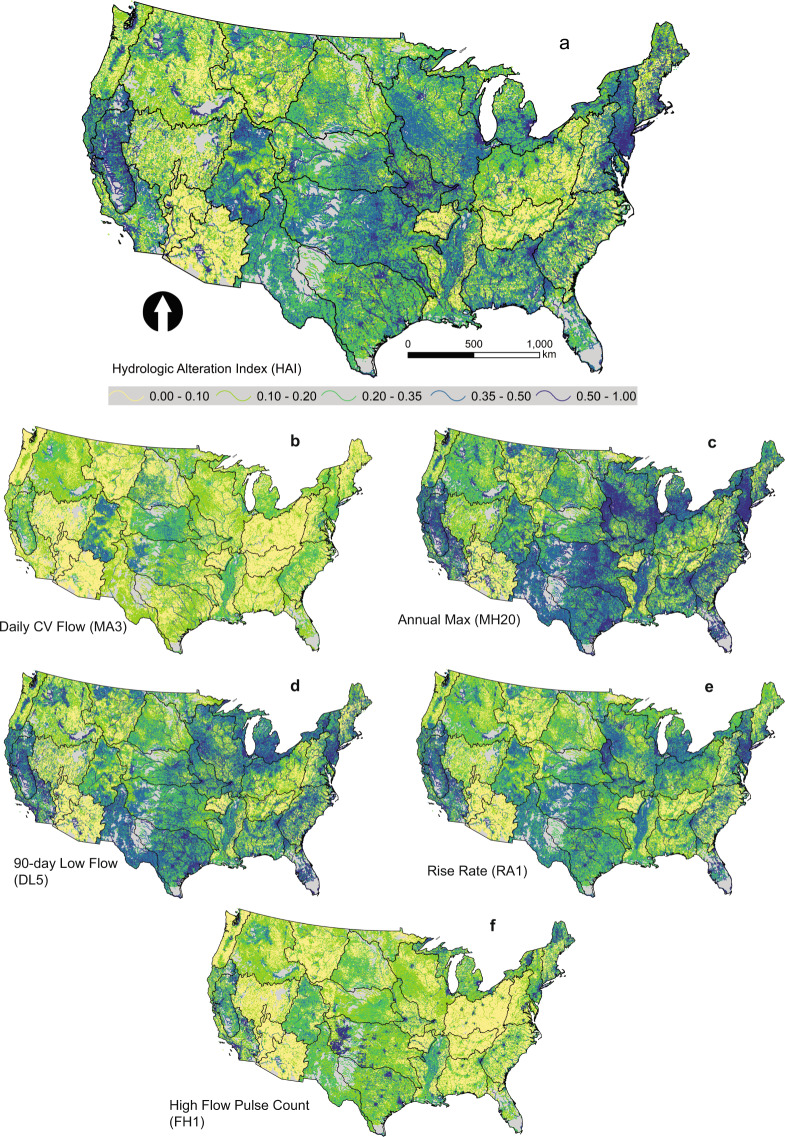
Six examples of hydrologic alteration indices mapped to stream reaches. (**a**) Hydrologic alteration index (HAI), (**b**) Daily CV Flow (MA3), (**c**) Annual Max divided by catchment area (km2) (MH20), (**d**) 90-day Low Flow (DL5), (**e**) Rise Rate (RA1), and (**f**) High Flow Pulse Count (FH1). See Table 1 for details on hydrologic index descriptions. Regional boundaries represent ecohydrologic regions.

Random forest models were constructed to predict each hydrologic alteration index at stream gages using an ensemble of human disturbance variables summarized in the upstream basins contributing to each gage. Predictor variables included landcover, dam storage, infrastructure, and water withdrawals (Supplementary Table 1). Random forest models were developed for the entire US and for each of 29 ecohydrologic regions (Fig. 2), which represent unique combinations of Freshwater Ecoregions^29^ and two-digit hydrologic unit codes (i.e., major river basins). The same human disturbance variables were compiled in the networks upstream of all NHDplus V1 stream reaches (https://nhdplus.com/NHDPlus/NHDPlusV1_home.php) and models were then applied to predict hydrologic alteration in those reaches. Values were then extended to NHDplus V2 stream reaches (https://www.epa.gov/waterdata/get-nhdplus-national-hydrography-dataset-plus-data) using crosswalk tables.

#### Step 6 and 7 – Predicting and mapping ecological responses to hydrologic alteration in stream reaches

Comprehensive maps of hydrologic alteration in stream reaches provide a foundation for subsequent modeling efforts, such as evaluating ecological responses to altered streamflow. Once these flow-ecology relationships are developed for multiple regions, ecological conditions, similar to hydrologic conditions, can be extrapolated to stream reaches. Recently, George *et al*.^17^ used the same hydrologic alteration indices reported in this study to develop regionally explicit nationwide flow-alteration-ecological-response relationships. These models were used to extrapolate ecological conditions (i.e., losses in fish species richness) to the stream reach resolution based on modeled hydrologic alteration values.

### Step 3 expanded: Calculating hydrologic alteration indices for stream gages

We elaborate on the above methodology, starting here with step 3. We used two approaches for calculating hydrologic alteration for non-reference stream gages^24^. For the majority of indices (Table 1), we calculated hydrologic alteration as proportional changes of observed (O) index values (i.e., human-altered conditions), versus expected (E) index values (i.e., reference conditions) in the equation: (O – E)/E^30^. Hence, indices ranged from −1 to values» 1. Performance, as measured by area-under-the-curve (AUC), of preliminary models using these raw values were lower than desired (AUC < 0.7). Hence, following Eng *et al*.^30^, we scaled values from 0 to 1 to represent a likelihood of hydrologic alteration in the following fashion. For indices < = 1, we used the absolute value of (O – E)/E, whereas indices >1 were assigned maximum values of 1. For reference gages, hydrologic alteration values were set to 0 for each metric.

While the above measures are informative for individual flow components, indices that summarize the multi-dimensional nature of stream flow alterations provide convenient single measures of alteration. We calculated a seasonality index, analogous to Zaerpour *et al*.^7^, representing shifts in the monthly flow magnitudes, as cumulative differences in observed (O) and expected (E) values for all mean monthly flows using the following equation:

1 $$\mathop{\sum }\limits_{m=i}^{12}\left(\frac{\left({O}_{i}-{E}_{i}\right)}{{E}_{i}}\right)$$

As a second multidimensional measure, we calculated a cumulative hydrologic alteration index (HAI), which evaluates the degree of separation between the flow regime of non-reference streams’ and that of reference streams within the same hydrologic class. Hydrologic classes represent groups of streams that share similar natural hydrologic patterns. McManamay *et al*.^23^ developed a hydrologic classification of reference streams in the US, and subsequently, non-reference gages were assigned to those hydrologic classes using models^22^. To calculate the HAI, all 110 hydrologic metrics (step 2 above) for reference and non-reference gages were centered, scaled from 0 to 1, and assessed in a principal components analysis (PCA)^24^. Thirteen of the components were significant according to the broken-stick method^31^. We partitioned the 13 principal component scores by hydrologic class membership and calculated 90^th^ percentile confidence intervals for only reference streams. The confidence interval (*a…b*) for significant PC scores (*S*) is represented by the lower (*a*) and upper (*b*) bounds. For each non-reference gage and each significant PC, we calculated a rank (*r*) value using the following:

2 $${\rm{If}}\;{a}_{i}\le {S}_{i}\le {b}_{i}\;{\rm{is}}\;{\rm{true}}\;{\rm{then}}\;{r}_{i}=0,\;{\rm{otherwise}}\;{r}_{i}={V}_{i},$$

Where *V*_*i*_ is the eigenvalue for the *i*^*th*^ significant PC. The HAI was then calculated for each non-reference gage using:

3 $${\sum }_{i=1}^{n}\left|{S}_{i}-{a}_{i}\right|\ast {r}_{i}\;{\rm{for}}\;{S}_{i} < {a}_{i},{\rm{and}}\;{\,\sum }_{i=1}^{n}\left|{S}_{i}-{b}_{i}\right|\ast {r}_{i}\;{\rm{for}}\;{S}_{i} > {b}_{i}$$

The formula accounts for both the degree of alteration of the PC (i.e., *S*_*i*_*-a*_*i*_ or *S*_*i*_*-b*_*i*_) as well as the importance of each PC to overall variability in hydrologic regimes (i.e., eigenvalue, *V*_*i*_).

To ensure all metrics were on a similar scale, both the seasonality index and HAI were scaled from 0 to 1 for each ecohydrologic region based on:

4 $$\frac{{x}_{i}-{\rm{\min }}(x)}{\max \left(x\right)-{\rm{\min }}(x)}$$

### Step 4 and 5 expanded: Hydrologic alteration models and mapping

Random forests^32^ were constructed to model all hydrologic alteration indices as binomial distributions using 50 predictor variables summarizing natural characteristics and human disturbances, such as landscape alteration and infrastructures (Supplementary Table 1, see^24^). Random forests are a form of machine learning where large numbers of decision trees are constructed in an iterative fashion using a bootstrapped subsample of observations and subsets of variables^32^. The remaining observations are termed the out-of-bag (OOB) sample, which is used in cross-validation measurements of variance explained, error, and variable importance. Each tree is constructed from training data and then predictions are combined among all trees. We used the randomForest package^33^ in the R programming environment to develop tree-based models for the entire US (all gauges) and separately for each ecohydrologic region. Hence, with 43 hydrologic indices and 29 regions, over 1,000 forest models were generated. Predictor variables used in models are classified into 8 groups (number of variables in parentheses): urbanization (14), agriculture (10), dams and reservoirs (6), power generation (6), dischargers and flow modifiers (5), human disturbance indices (3), basin size, stream size, and climate (3), and natural land cover (3) (Supplementary Table 1). Predictor variables were obtained from multiple sources or our own geospatial analysis and were summarized for both the local catchment surrounding each stream reach containing the stream gauge or were accumulated for the entire catchment contributing to each gauge (Supplementary Table 1). Similarly, the same predictor variables were compiled for all 2.6 million NHDPlus V1 stream reaches, both for local catchments and entire stream networks upstream of each reach. Following construction and calibration, random forest models were used to extrapolate hydrologic alteration values to all 2.6 million NHDPlus V1 stream reaches in the CONUS (Fig. 2). Using crosswalks between NHDPlus V1 and V2, we extended hydrologic alteration values to NHD Plus V2 stream reaches.

### Step 6 and 7 expanded: Ecological alteration models and mapping

To develop flow-alteration-ecological response relationships, George *et al*.^17^ developed a comprehensive dataset of overlapping hydrologic and ecological data for 6,452 stream reach locations. At each location, measures of hydrologic alteration and ecological alteration were compiled, where ecological alteration was measured as the deviation in observed native fish richness from expected natural conditions^17^. Flow-alteration-ecological-response relationships typically adopt a “wedge-shaped” distribution well-suited for quantile regression^34^. Hence, George *et al*. generated quantile regression models predicting 50^th^, 75^th^, and 95^th^ percentile alterations in fish richness from hydrologic alteration values for all hydrologic metrics, except HAI, within each 4-digit hydrologic unit code (HUC-4), except watersheds where limited sample size prohibited model development (12% of watersheds).

**Fig. 3:**
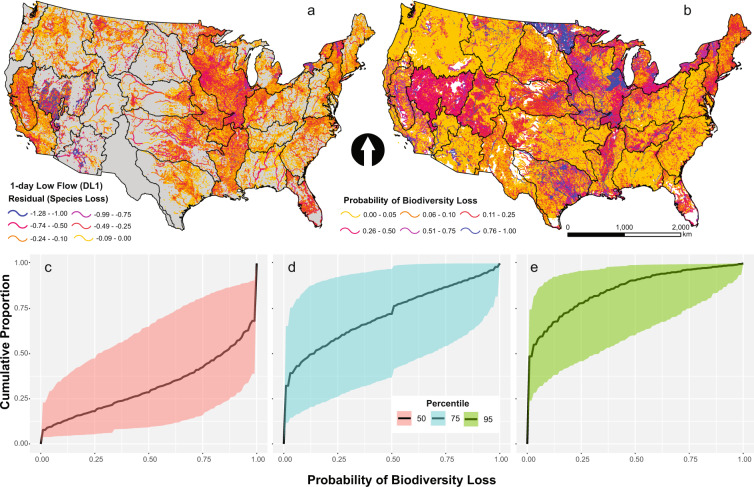
Modeled fish biodiversity responses to hydrologic alteration and assessment of uncertainty. (**a**) Losses in fish species richness (measured as residuals) estimated in response to alterations in 1-day low flows (DL1). Fish residuals were estimated using the 95^th^ quantile regression. (**b**) Probability of fish biodiversity loss estimated from fish richness responses to 42 hydrologic alteration metrics. Fish biodiversity losses were estimated using the 95^th^ quantile regressions for all metrics. (**c**–**e**) Cumulative proportions of stream reaches exceeding a given probability of fish biodiversity loss based on (**c**) 50^th^, (**d**) 75^th^, and (**e**) and 95^th^ quantile regressions. Ranges for each cumulative distribution represent compounded uncertainty arising from error in hydrologic alteration models and quantile regressions.

Quantile regression model coefficients for each hydrologic metric within each HUC4 were used to predict alterations in native fish richness at the stream reach resolution based on modeled estimates of hydrologic alteration. In situations where coefficients were unavailable for HUC-4s, average model coefficients for entire ecohydrologic regions were used. Residuals in fish richness were calculated for each hydrologic alteration metric (e.g., Fig. 3a). Flow thresholds or tipping points represent hydrologic alteration values beyond which ecological degradation is expected^13^ or can be deemed socially unacceptable^15^. Presuming that loss of any native fish species is unacceptable, the hydrologic alteration value at which residuals in fish richness <0 is considered the threshold or limit. Based on the quantile regression models^17^, thresholds were identified and applied to all stream reaches based on HUC-4 or ecohydrologic region. Modeled hydrologic alteration values for each metric were compared to each respective threshold in each stream reach to yield a binary response where losses in fish biodiversity are expected (1) or not (0), depending on if the hydrologic threshold was exceeded. The mean value among these responses for all hydrologic alteration metrics yields a probability of fish biodiversity loss (Fig. 3b), ranging from 0 to 1, based on all components of the flow regime.

## Data Records

All data records available and their file structure are described in Table 2. Modeled estimates of hydrologic alteration for all 43 metrics and estimates of fish biodiversity loss in response to each metric (except HAI) are provided at the NHDPlus stream-reach resolution in .csv or .txt files. Each row entry includes the corresponding stream reach identifiers (i.e., COMIDs) for both NHDPlus V1 and V2 versions. Given that fish species richness responses were modeled for many hydrologic alteration metrics, the median and extreme fish biodiversity loss values across all metrics, in addition to binary and probability measures of fish biodiversity loss, are also provided for each stream reach. Estimates of hydrologic alteration are provided separately for different modeled outcomes, including the random forests developed for all gauges in the US and the aggregated results from all forests developed for each ecohydrologic region. Likewise, the fish species richness response data are provided in two datasets for reach set of responses, one for the entire US and one for the aggregated regional results. The data are generally split into two or four regional sub-datasets for the entire CONUS. Random forest model performance measures, including AUC values and relative importance of predictor variables are also provided in .csv files, both for the entire-US model and for each ecohydrologic region model. Data records are freely and openly accessible under a Creative Commons Attribution 4.0 International license on Zenodo^35^.

**Table 2:** Description of files in the Data Record openly accessible through Zenodo^35^.

File(s)	Description
Ecohydrologic_Regions	ESRI shapefile of regions used for separate hydrologic alteration model development
Hydrologic_alteration.zip	Predicted values of hydrologic alteration in 43 metrics at the stream reach resolution. Predicted values were estimated using regional models or models developed for the entire US. Stream reach identifiers are provided for NHDPlus V1 and V2 stream reaches.
Model_peformance_AUC.zip	Performance, as measured by Area-under-the-curve (AUC), of regional models and US-wide models in predicting hydrologic alteration values. Two measures of performance were used: 1) the ability of the model to differentiate hydrologic alteration between reference and non-reference streams and 2) the ability of the model to predict hydrologic alterations greater than and less than 0.5.
Variable_importance_RF.zip	The importance of variables used as predictors of hydrologic alteration across all hydrologic metrics and models. Variable importance is provided for each regional model and for the entire US model for all metrics.
Fishresponses_allmetrics.zip	Estimates of fish richness responses to hydrologic alteration for 43 hydrologic metrics. Values are provided for each hydrologic metric and each stream reach. Responses are modeled using 50th, 75th, and 95th quantile regression models. Hydrologic alteration values were generated for both regional models and US models.
Fish_Responses_biodiversity_loss_prob.zip	Probabilities of fish biodiversity loss in each stream reach based on 50th, 75th, and 95th quantile regression models (mean, minimum and maximum values) for all hydrologic alteration metrics. Two files are provided, one based on probabilities of biodiversity loss using predicted hydrologic alteration values from region-specific models (ecohydrologic regions) and for models developed for the entire US.
Fish_Responses_min_median_rich_delta.zip	Estimated minimum and median changes in fish richness (or delta fish richness) in each stream reach in response to hydrologic alteration across all 43 hydrologic metrics. Separate fish responses were developed for 50th, 75th, and 95th quantile models evaluating fish species response to hydrologic alteration. Additionally, separate analyses were conducted from region-specific hydrologic alteration models and models developed for the entire US.

## Technical Validation

We evaluated hydrologic alteration model performance using area-under-the-curve (AUC) measures for all models. AUC values represent cross-validation measures, as predictions from model trees are taken from the out-of-bag (OOB) sample, representing roughly 33% of observations, modeled in that forest. AUC values are appropriate for evaluating the performance of hydrologic alteration models as the indices range from 0 to 1; however, AUC evaluation requires a binary classifier as the control from which to measure performance. In this case, we used two binary control measures to calculate AUC values: a binary categorization of reference and non-reference gages (measure 1), and a categorization of high (index > 0.5) and low (index <  = 0.5) hydrologic alteration values in the empirical observations (measure 2). Relative importance (RI) of variables in predicting hydrologic alteration indices was also measured from the OOB sample as the average decrease in mean squared error (MSE) rates across all permutations of a given variable among all trees. Because RI can vary with MSE values, we scaled all RI values from 0 to 1 using the Eq. 4 to support comparison among hydrologic alteration indices, groups of predictor variables, and regions. MSE values for all random forest models are provided in Supplementary Material 1.

**Fig. 4:**
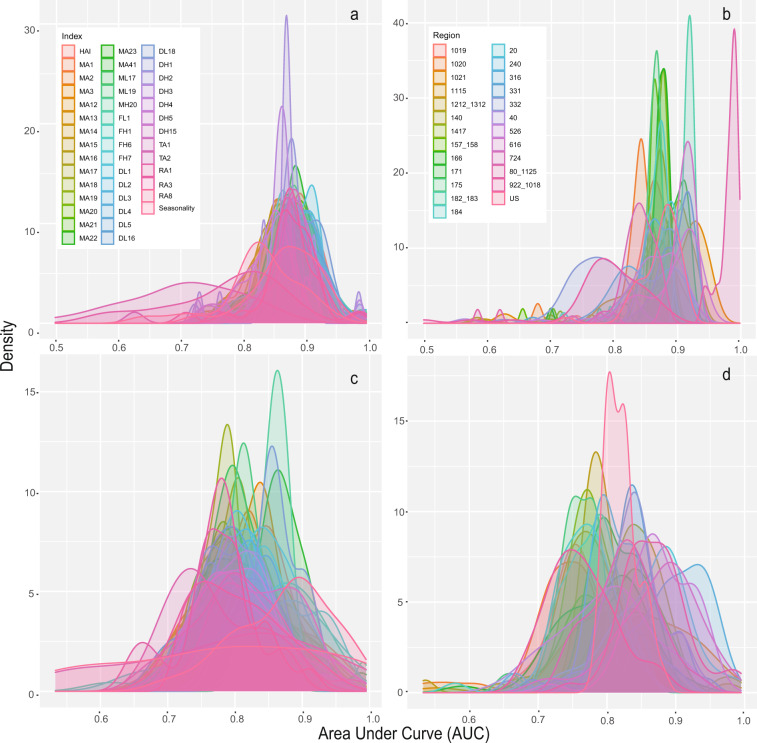
Area Under the Curve (AUC) values assessment random forest model performance using two measures. Density plots display the frequency of AUC values measuring model performance for distinguishing (**a**) reference and non-reference gauges (measure 1) for all hydrologic indices, and for (**b**) all ecohydrologic regions, and distinguishing observed hydrologic alteration values > or <0.5 (measure 2) for (**c**) all hydrologic indices, and (**d**) all ecohydrologic regions.

AUC values for models developed for the entire US averaged 0.87 and ranged from 0.72 to 0.91 for measure 1, whereas for measure 2, AUC values averaged 0.82 and ranged from 0.78 to 0.88 (Fig. 4, results also available via^35^). Performance for models developed for individual ecohydrologic regions varied considerably. For measure 1, AUC values averaged 0.87 and ranged from 0.5 to 0.998 depending on the hydrologic index (Fig. 4a) and region (Fig. 4b). For measure 2, AUC values averaged 0.81 and ranged from 0.53 to 0.995 (Fig. 4c,d). Variation in model performance seemed to be related to intrinsic properties of each region and less influenced by different hydrologic indices. Differences in model performance was not influenced by the gauges available for model construction. For instance, there was no relationship between the number of observations (number of gauges) and average variation explained in hydrologic indices for each ecohydrologic region.

**Fig. 5:**
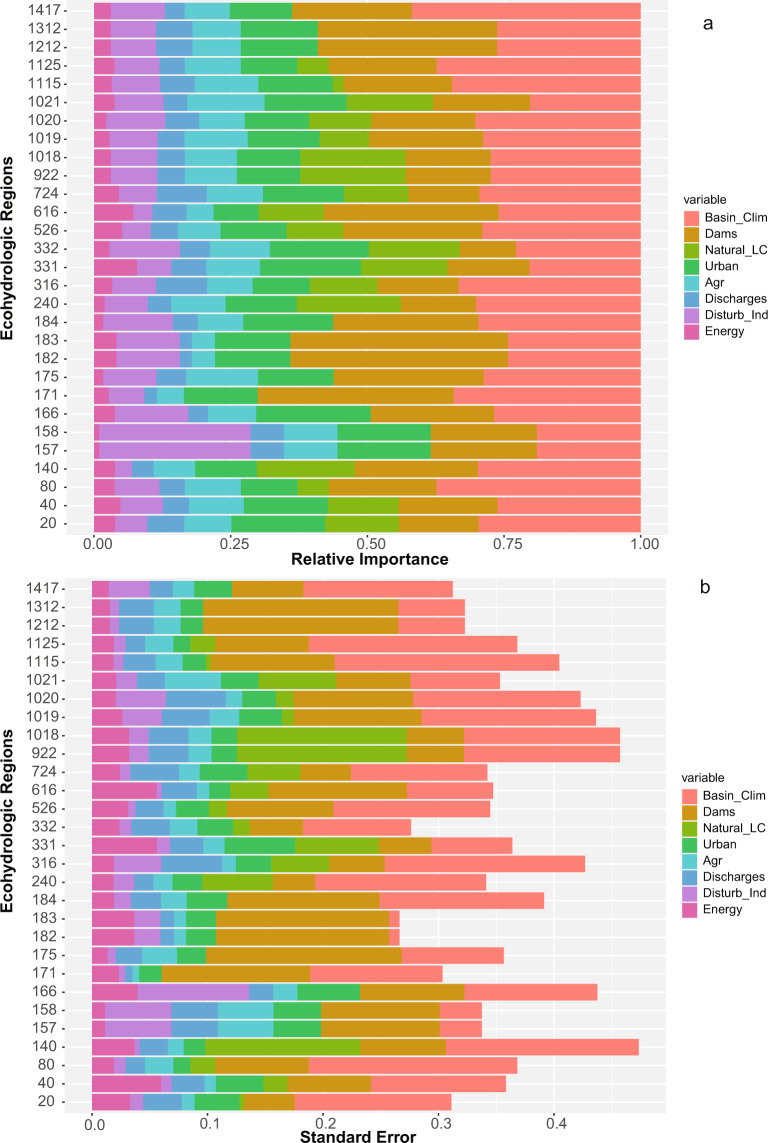
Relative importance of variables in hydrologic alteration models. (**a**) Relative importance values for predictors were grouped within variable types (e.g., basin, dams) and then averaged with the group and across all models within a region. (**b**) standard error in relative importance values within a group and region.

The relative importance of predictor variables in explaining variation in hydrologic indices was also explored. Climate and natural basin characteristics, such as drainage area and precipitation, were the most important predictor variables, followed by variables characterizing dams (e.g., dam storage, degree of regulation). Landcover variables were of moderate importance, whereas energy infrastructure and water discharges (unrelated to dam regulation) were of minor importance (Fig. 5a). With a few exceptions, variation in importance among models within a region generally mirrored the overall importance of variables, where basin and climate variables had higher variation in importance, followed by dam-related variables (Fig. 5b).

As a second and coarse validation of hydrologic alteration, we compared our nationwide rates of hydrologic alteration from our assessment to three other nationwide assessments of hydrologic alteration, conducted at either the stream-gage or stream-reach level^8,26,36^. Comparing rates of alteration among different assessments requires establishing common hydrologic alteration thresholds, which, in the case of Carlisle *et al*.^8^, was > = 10% change in hydrologic indices from reference conditions. Carlisle *et al*. found that 86% of stream gages showed signs of at least 10% deflated mean annual minima and maxima values. Based on our assessment, 93% of stream gages had alteration values of > = 0.1 for DL1 (1-day annual minimum), 66% of gages with > = 0.1 for DH1 (1-day annual maximum), and 96% of gages considering both DL1 and DH1. In contrast to Carlisle *et al*., our analysis considers both decreases and increases to hydrologic metrics as a measure of alteration. Extending the comparison to stream reaches, approximately 74% of U.S. stream mileage, on average (79% median), across all hydrologic indices had hydrologic alternation values equal or exceeding 10%. According to the hydrologic alteration index (HAI), 79% of streams were hydrologically altered, whereas the mean daily flow index (MA1) indicated 81% of streams were altered.

**Fig. 6:**
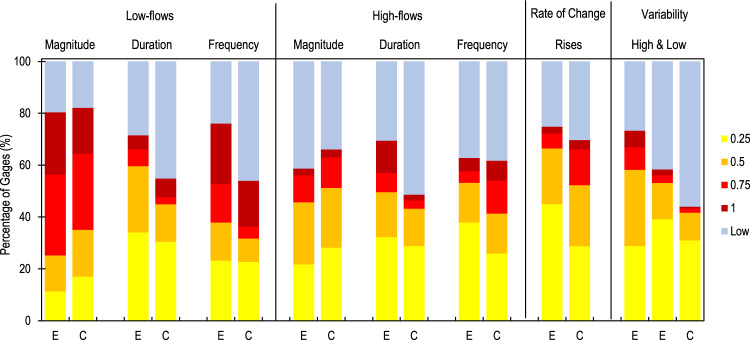
Comparison of hydrologic alteration assessments of stream gauges conducted by Eng *et al*. (2019) (E) and that of the current study (C). Percentages of stream gages having various degrees of hydrologic alteration (colors) are compared between the two studies. Analogous hydrologic statistics were selected for comparison in each category (e.g., Low-flows magnitude); however, no statistics were exactly the same between the two studies and likely contributed to differences.

Eng *et al*.^26^ conducted a thorough study of hydrologic alteration at stream gages and reported percentages of non-reference gages displaying alteration for twelve hydrologic metrics, some of which were compatible with those used in our study, although none were identical (Supplementary Material 2, Table S1). Aside from differences in the metrics used, the scale of values used in Eng *et al*.’s approach to measure degrees of hydrologic alteration differed from our approach and required some adjustment to be compatible for comparison (Supplementary Material 2, Table S2). Generally, the frequency of non-reference stream gages displaying degrees of alteration for various flow components in our study agreed relatively well with that of Eng *et al*. (Fig. 6). Carlisle *et al*.^36^ used machine learning models to extend Eng *et al*.’s stream gage assessment to the stream reach level and reports that 1.2 million km, or roughly 38% of streams have “modified flows”. Carlisle et. al. does not report what threshold was used to generate this result; however, if we assume that a 20% presumptive standard threshold^13^ was used, then our dataset suggests 57% of streams are hydrologically altered based on HAI values > = 0.2 (note: HAI is a more comprehensive measure of 110 hydrologic metrics, compared to the 12 reported in Eng *et al*.). Collectively, despite inconsistencies in approaches, these comparisons suggest that our hydrologic alteration assessment is within reasonable estimates of other nationwide assessments.

Validation of the ecological alteration data required consideration of compounding error arising from both the hydrologic alteration models and quantile regression models. Hence, cumulative uncertainty in fish biodiversity loss can stem from error in the quantification of hydrologic alteration thresholds, as well as estimation of hydrologic alteration. Using mean-square error (MSE) rates for each random forest model, modeled hydrologic alteration estimates $(\hat{HA})$ were adjusted for each stream reach by subtracting or adding MSE values (from random forest models), yielding minimum $(\overline{HA})$ and maximum $(\overline{\overline{HA}})$ values, respectively, for each hydrologic metric. For quantile regression models, standard error values for slope coefficients were used to vary slopes to yield minima $(\bar{T})$ and maxima $(\overline{\overline{T}})$ threshold values for each hydrologic metric. Subsequently, fish biodiversity loss (*R*) was modeled as a binary outcome of surpassed thresholds for the *h*^*th*^ hydrologic metric at the *i*^*th*^ stream reach, where

5 $${R}_{h,i}\in \left\{0,1\right\}{\rm{for}}\;{\rm{all}}\;h\;{\rm{and}}\;i.$$

Lower limits (${\bar{R}}_{h,i}$) and upper limits $({\overline{\overline{R}}}_{h,i})$ of fish biodiversity loss were subject to the following constraint:

6 $${\rm{If}}\,{\bar{T}}_{h}\ge {\overline{\overline{HA}}}_{h,i},\;{\rm{then}}\;{\overline{R}}_{h,i}=1,\;{\rm{else}}\;{\overline{R}}_{h,i}=0,$$

and

7 $${\rm{If}}\,{\overline{\overline{T}}}_{h}\ge {\overline{HA}}_{h,i},\;{\rm{then}}\;{\overline{\overline{R}}}_{h,i}=1,\;{\rm{else}}\;{\overline{\overline{R}}}_{h,i}=0.$$

Lower and upper limits to the probability of fish biodiversity loss for each stream reach were then calculated as:

8 $$\frac{{\sum }_{h}^{n}{\bar{R}}_{h,i}}{n}$$

and

9 $$\frac{{\sum }_{h}^{n}{\overline{\overline{R}}}_{h,i}}{n}$$

Where n is the number of hydrologic metrics. Lower and upper limits of probability of fish biodiversity loss were calculated separately for 50^th^, 75^th^, and 95^th^ quantile model results, each of which had different hydrologic threshold values corresponding to each hydrologic alteration metric.

Cumulative frequency distributions were used to examine the variability and uncertainty in the probability of fish biodiversity loss in stream reaches according to different quantile models. The distribution of fish biodiversity loss probability varied widely among different quantiles (Fig. 3c–e). Uncertainty was considerable, indicating high amounts of compounding error arose from both hydrologic alteration models and quantile models predicting fish biodiversity loss (Fig. 3c–e); however, total uncertainty was lower for the 95^th^ percentile models (Fig. 3e).

## Usage Notes

Mapping hydrologic alteration indices and associated measures of ecological responses in stream reaches provides a rich dataset for environmental flow applications, especially in data-poor regions. The dataset provides general “rules of thumb” for environmental flow guidelines, specifically which hydrologic indices are important to ecology, and hence, management. Maps at the stream-reach resolution can be used to explore hydrologic indices with the highest rates of alteration, as well as the risk of stream habitat alteration and biodiversity loss. As such, the data can be useful for exploring the frequency of alteration in the landscape according to hydrologic or biologically meaningful spatial units (e.g., species ranges), and can support aquatic species GAP analyses or prioritization for areas of flow restoration. Data are provided in accessible .csv formats and summarized at the NHDplus stream-reach scale. Hence, the data provide inherent interoperability with other NHDplus-derived products with associated COMID identifiers. The full functionality of the NHDplus framework, including topological connections and stream routing, can be leveraged with the hydrologic alteration dataset.

Users of the dataset should be aware of its limitations. The modeled hydrologic alteration and ecological alteration values are not meant to replace site-specific environmental assessments and are most appropriately used as information building blocks for regional environmental flow applications. As indicated in the validation section, random forest models of hydrologic alteration had variable error rates. While model performance is satisfactory for most hydrologic metrics, uncertainty would have likely reduced had we controlled for climate variation among reference and non-reference gages^26^. Although 15 years of hydrologic data records is considered sufficient for calculating hydrologic statistics^25^, a formal control of climate shifts would have likely improved model performance. Furthermore, compounded uncertainties were significant when predicting fish biodiversity responses to alteration. An important consideration is the interpretation of hydrologic alteration values, which have been standardized from 0 to 1. As such, these are not directly translatable as measures of hydrologic alteration. Although values were generally derived from ratios of alteration and intended to represent a probability of alteration, interpreting the values as measures of risk and indicators of alteration is more appropriate than in a strict probabilistic sense. Finally, random forest models developed for each ecohydrologic region were influenced by the range of hydrologic alteration values and frequency of altered streams in those regions. Additionally, values were standardized based on minima and maxima within each ecohydrologic region; therefore, hydrologic alteration values derived from regional models will display slight regional biases in values. Therefore, users desiring US-wide applications should perhaps use values from US-wide models.

## Supplementary information

Supplementary Information 1

Supplementary Information 2

Supplementary Table 1

## Data Availability

Data processing, visualization, and random forest development were conducted using pre-existing libraries, particularly the randomForest package^33^ and ggplot2 package^37^ (for density plots and stacked bar plots) in the R programming environment, whereas spatial mapping was conducted in ArcMap 10.7.1, as indicated in the methods and validation. Therefore, no custom code was used for this study.
