# Supplementary material for: Mapping hydrologic alteration and ecological consequences in stream reaches of the conterminous United States
Source: Sci Data. 2022 Jul 28;9:450. doi: 10.1038/s41597-022-01566-1 (PMC9334386; doi:10.1038/s41597-022-01566-1)
Supplement: Supplementary file 1 — Supplementary Information 1 [file 41597_2022_1566_MOESM1_ESM.pdf]

Supporting Information 1 – Random Forest Model Performance- Mean Square Error

Mapping Hydrologic Alteration and Ecological Consequences in Stream Reaches of the Conterminous United States

Ryan A. McManamay[1]*, Rob George[2], Ryan R. Morrison[3], Benjamin L. Ruddell[2]

[1]Department of Environmental Science, Baylor University, Waco, TX, USA 76798

[2]School of Informatics, Computing and Cyber Systems, Northern Arizona University, Flagstaff, AZ, USA 86011

[3]Department of Civil and Environmental Engineering, Colorado State University, Fort Collins, CO, USA 80523

Table of Contents:

Figure S1. Mean square error (MSE) for random forest models among all hydrologic metrics. Density plots display the frequency of MSE values measuring model performance for predicting hydrologic indices, ranging from 0 to 1.

Figure S2. Mean square error (MSE) for random forest models among all ecohydrologic regions. Density plots display the frequency of MSE values measuring model performance for predicting hydrologic indices, ranging from 0 to 1.

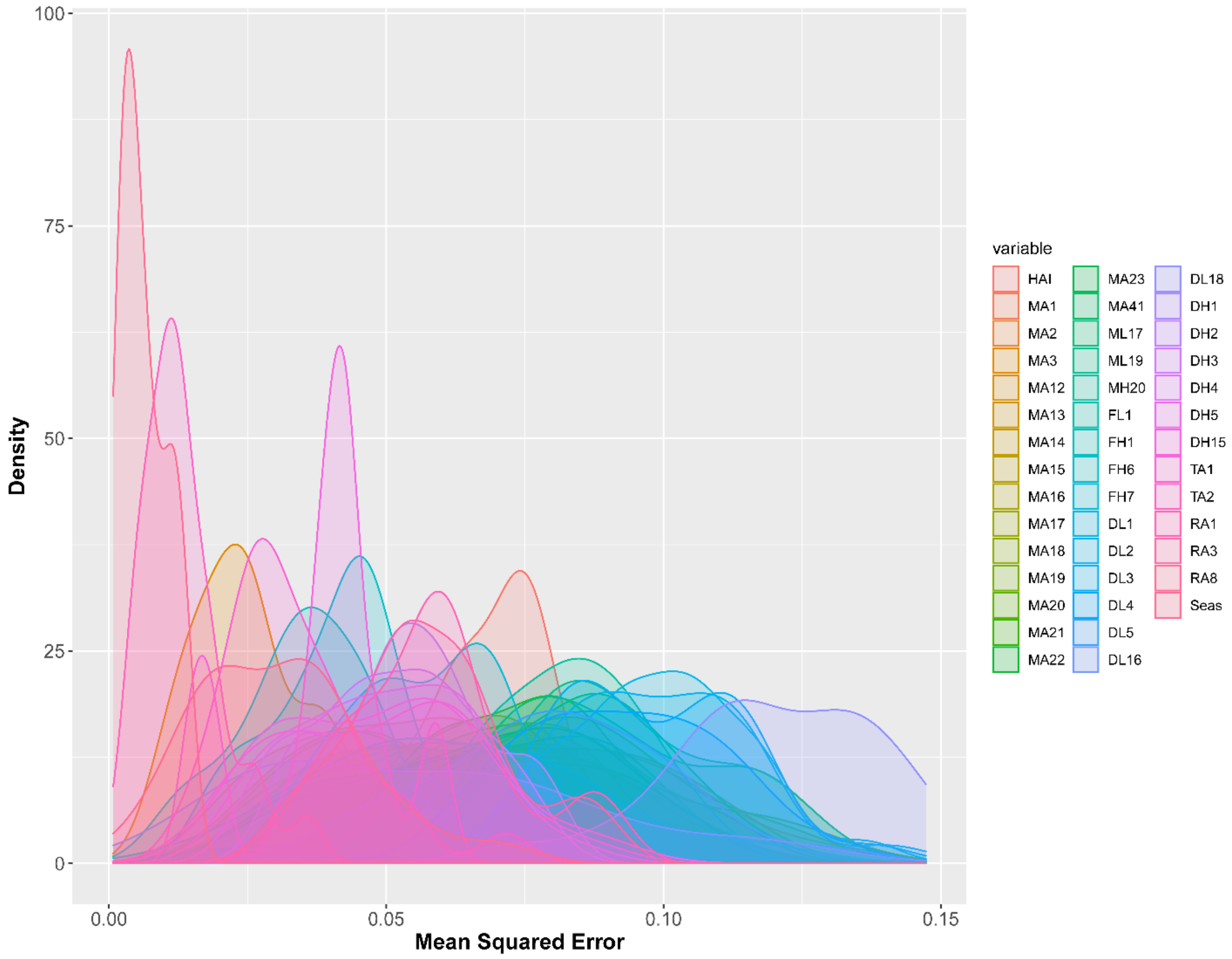

Figure S1. Mean square error (MSE) for random forest models among all hydrologic metrics. Density plots display the frequency of MSE values measuring model performance for predicting hydrologic indices, ranging from 0 to 1.

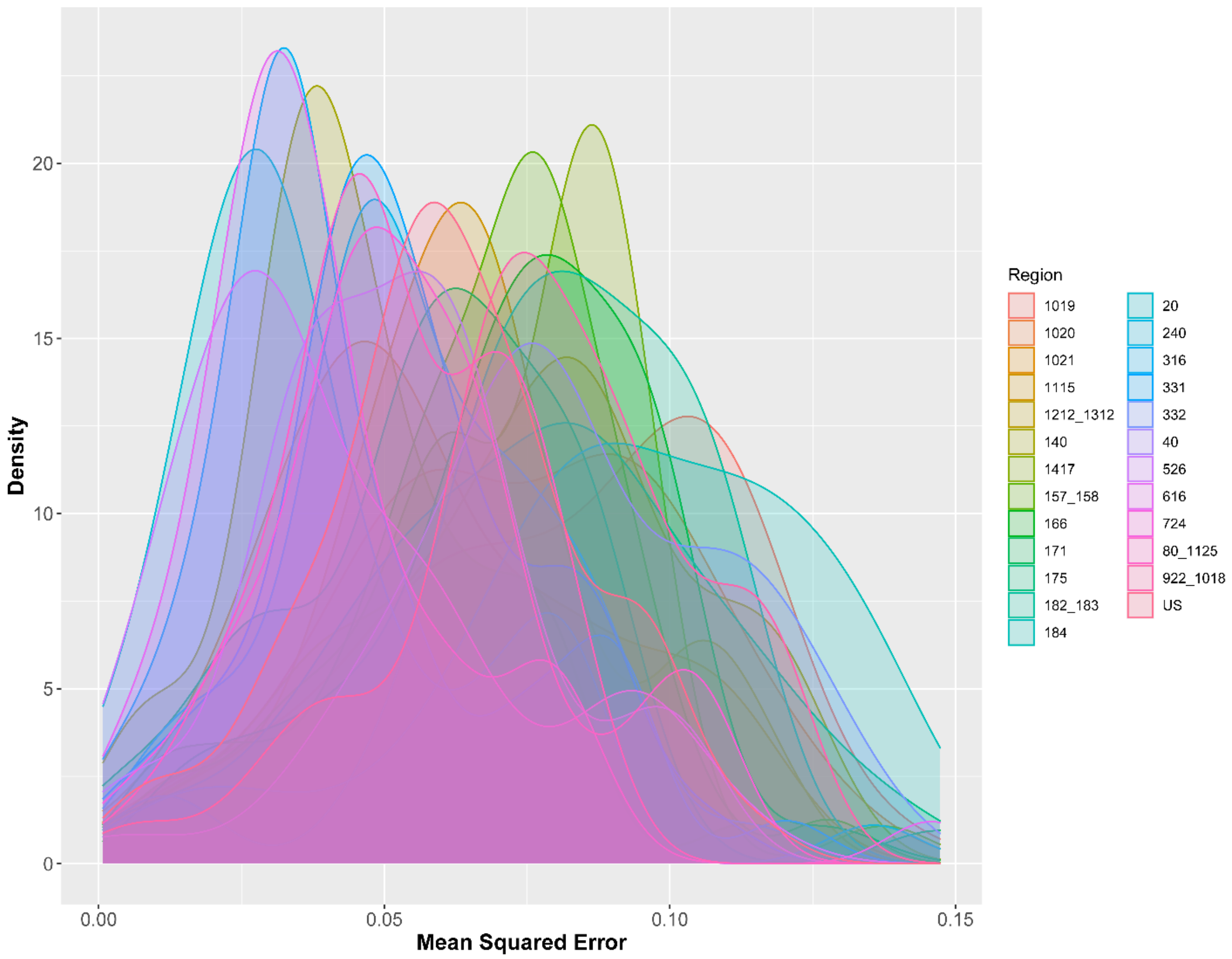

Figure S2. Mean square error (MSE) for random forest models among all ecohydrologic regions. Density plots display the frequency of MSE values measuring model performance for predicting hydrologic indices, ranging from 0 to 1.
