# Supplementary material for: Mapping hydrologic alteration and ecological consequences in stream reaches of the conterminous United States
Source: Sci Data. 2022 Jul 28;9:450. doi: 10.1038/s41597-022-01566-1 (PMC9334386; doi:10.1038/s41597-022-01566-1)
Supplement: Supplementary file 2 — Supplementary Information 2 [file 41597_2022_1566_MOESM2_ESM.pdf]

Supporting Information 2 – Comparison to Eng et al. (2019)

Mapping Hydrologic Alteration and Ecological Consequences in Stream Reaches of the Conterminous United States

Ryan A. McManamay[1]*, Rob George[2], Ryan R. Morrison[3], Benjamin L. Ruddell[2]

[1]Department of Environmental Science, Baylor University, Waco, TX, USA 76798

[2]School of Informatics, Computing and Cyber Systems, Northern Arizona University, Flagstaff, AZ, USA 86011

[3]Department of Civil and Environmental Engineering, Colorado State University, Fort Collins, CO, USA 80523

Table of Contents:

Extended Methods – Provides background of Eng et al. (2019) and our comparison

Table S1. Hydrologic metrics used by Eng et al. (2019) and analogous hydrologic metrics used as a comparison in the current study. Metric codes from the current study are provided in Table 1 in the main manuscript. P01 is the 1st-percentile non-exceedance flow or the 1% of daily flows that are less than the value. P10 and P90 are similar and represent 10th and 90th non-exceedance flows.

Table S2. Degrees of hydrologic alteration from Eng et al. and corresponding value in the current study.

Extended Methods:

Eng et al. (2019) conducted a hydrologic alteration assessment of stream gages very similar to the current study. The hydrologic metrics used and the approach to reporting degrees of alteration differed however between Eng et al.'s study and our approach. Table S1 provides the 12 hydrologic metrics used by Eng et al. (2019) and analogous hydrologic metrics for comparison used by the current study.

Eng et al. (2019) reported hydrologic alteration as a raw ratio of observed hydrologic metrics (O_{HM}) to the expected hydrologic metrics (E_{HM}), which is different than the calculation approach we used. Therefore, Eng et al.'s degrees of hydrologic alteration ranged from 0 to >2, whereas our values ranged from 0 to 1. To be compatible with our study, we translated Eng et al.'s hydrologic alteration values into a scale compatible with ours. For instance, in Eng et al., a hydrologic alteration value of 0.25 for a low-flow magnitude metric indicates that flows are only 25% of the expected magnitude, or 75% deflated. This would translate to a value of 0.75 in our analysis. However, a value of 1.25 in Eng et al., corresponds to a 25% inflation in streamflow relative to the expected condition. In our analysis, this value would likewise translate to 0.25. Table S2 below provides corresponding values between the two approaches.

Table S1. Hydrologic metrics used by Eng et al. (2019) and analogous hydrologic metrics used as a comparison in the current study. Metric codes from the current study are provided in Table 1 in the main manuscript. P01 is the 1st-percentile non-exceedance flow or the 1% of daily flows that are less than the value. P10 and P90 are similar and represent 10th and 90th non-exceedance flows.

**Eng et al. Hydrologic metric**	**Description**	**Current Study Hydrologic Metric**
	**Low flows**	
Magnitude	P01/DA	DL1
Variability	CV of annual minimum daily flows	MA3
Frequency	Average annual number of flow pulses < P10	FL1
Duration	Average annual duration of flow pulses <P10	DL16
Timing/seasonality	Seasonal distribution of flows <P10	Not available
	**High flows**	
Magnitude	P99/DA	DH1
Variability	CV of annual maximum daily flows	MA3
Frequency	Average annual number of flow pulses >P90	FH7
Duration	Average annual duration of flow pulses >P90	DH15
Timing/seasonality	Seasonal distribution of flows >P90	Not available
	**Flow symmetry and stochasticity**	
Skew		Not available
Daily rises	Number of days where flow > previous day/ total number of days	RA1

Table S2. Degrees of hydrologic alteration from Eng et al. and corresponding value in the current study.

Direction of Alteration	Eng et al.	Current study
Diminishment	0.25	0.75
Diminishment	0.5	0.5
Diminishment	0.75	0.25
No Change	1	0
Inflation	1.25	0.25
Inflation	1.5	0.5
Inflation	1.75	0.75
Inflation	2	1
Inflation	>2	1
