# Supplementary material for: Mapping hydrologic alteration and ecological consequences in stream reaches of the conterminous United States
Source: Sci Data. 2022 Jul 28;9:450. doi: 10.1038/s41597-022-01566-1 (PMC9334386; doi:10.1038/s41597-022-01566-1)
Supplement: Supplementary file 3 — Supplementary Table 1 [file 41597_2022_1566_MOESM3_ESM.pdf]

Supplementary Table 1

Mapping Hydrologic Alteration and Ecological Consequences in Stream Reaches of the Conterminous United States

Ryan A. McManamay[1]*, Rob George[2], Ryan R. Morrison[3], Benjamin L. Ruddell[2]

[1]Department of Environmental Science, Baylor University, Waco, TX, USA 76798

[2]School of Informatics, Computing and Cyber Systems, Northern Arizona University, Flagstaff, AZ, USA 86011

[3]Department of Civil and Environmental Engineering, Colorado State University, Fort Collins, CO, USA 80523

Table 2. Predictor variables and their descriptions used in random forest models to predict hydrologic alteration values. Variables preceded by L indicate values are summarized only for the local NHDPlus catchment; N indicates the variable is summarized for the entire upstream network.

Variable	Description
ECOHYDR	Ecohydrologic regions (combination of freshwater ecoregions and HUC2 regions)
DRAIN_SQKM[1]	Upstream drainage area of stream reach in km^2
MAFLOWU[1]	Mean annual flow (m^3s^{-1})
PPT30MEAN[1]	30-y average precipitation averaged for entire upstream network
nidStorSQK	Cumulative sum of US Army Corps of Engineers National Inventory of Dams storage (megaliters) divided by upstream drainage area (km^2)
DOR	Degree of regulation (% of annual runoff stored by dams; see nidStorSQK)
N_prcnt_watr[1]	Percentage of water land cover
N_prcnt_brrn[1]	Percentage of barren land cover
N_prcnt_frst[1]	Percentage of forest land cover
N_prcnt_wetl[1]	Percentage of wetland land cover
L_prcnt_11[1]	Percentage of open water land cover
L_URBAN[2]	Percentage of low, medium, and high development intensity land cover
L_URBANL[2]	Percentage of low development intensity land cover
L_URBANM[2]	Percentage of medium development intensity land cover
L_URBANH[2]	Percentage of high development intensity land cover
L_AGR[2]	Percentage of all agricultural land cover types
L_PASTURE[2]	Percentage of pasture/hay land cover
L_CROPS[2]	Percentage of crop land cover
L_POPDENS[2]	Population density
L_ROADCR[2]	Road crossings per km^2
L_ROADLEN[2]	Road length (km) per km^2
L_DAMS[2]	Number of dams per km^2
L_NPDES[2]	Number of National Pollutant Discharge Elimination Systems (NPDESs) per km^2
N_URBAN[2]	Percentage of low, medium, and high development intensity land cover
N_URBANLC[2]	Percentage of low development intensity land cover
N_URBANMC[2]	Percentage of medium development intensity land cover
N_URBANHC[2]	Percentage of high development intensity land cover
N_AGR[2]	Percentage of all agricultural land cover types
N_PASTUREC[c]	Percentage of pasture/hay land cover
N_CROPSC[c]	Percentage of crop land cover
N_POPDENSC[c]	Population density
N_ROADCRC[c]	Road crossings per km^2
N_ROADLENC[2]	Road length (km) per km^2
N_DAMSC[2]	Number of dams per km^2

N_MINESC[2]	Number of mines per km^2
N_TRIC[2]	Number of Toxic Release Inventory Sites per km^2
N_NPDESC2[2]	Number of National Pollutant Discharge Elimination Systems (NPDESs) per km^2
N_CERCC[2]	Number of Superfund National Priorities List sites from the Compensation and Liability Information System
L_DistIndx[2]	Local Disturbance Index
NDistIndx[2]	Network Disturbance Index
CumDistInd[2]	Cumulative disturbance index, calculated from LDistIndx and NDistIndx
N_adrain_sum[3]	Cumulative area (m^2) subject to artificial drainage
N_irrig_sum[3]	Cumulative area (m^2) subject to irrigation
N_tiles_sum[3]	Cumulative area (m^2) of tile drains
N_ditch_sum[3]	Cumulative area (m^2) subject to the practice of ditches
N_mw_sum[4]	Sum of megawatt capacity of all power plants
N_mwh_sum[4]	Sum of megawatt hour generation of all power plants
N_Fdivmgd_sum[4]	Proportion of mean annual flow diverted for power plant generation
N_Fwthmgd_sum[4]	Proportion of mean annual flow withdrawn for power plant generation
N_Fconmgd_sum[4]	Proportion of mean annual flow consumed for power plant generation
N_Fsdivmgdsum[4]	Proportion of mean summer flow diverted for power plant generation

1. *Horizon Systems National Hydrography Dataset Plus*. NHDPlus Version 1 (Archive). https://nhdplus.com/NHDPlus/NHDPlusV1_home.php (2022)

2. *2010 NFHAP National Assessment of Fish Habitats* https://www.sciencebase.gov/catalog/item/5e31c23de4b0a79317d76bf3 (2022)

3. US Geological Survey. *Attributes for NHDPlus Catchments (Version 1.1) in the Conterminous United States: Artificial Drainage (1992) and Irrigation Types (1997)* https://water.usgs.gov/GIS/metadata/usgswrd/XML/nhd_adrain.xml (2021)

4. Energy Information Administration. *Form EIA-923 detailed data with previous form data (EIA-906/920)* https://www.eia.gov/electricity/data/eia923) (2022)
